# The Gluten-Free Diet for Celiac Disease: Critical Insights to Better Understand Clinical Outcomes

**DOI:** 10.3390/nu15184013

**Published:** 2023-09-16

**Authors:** Edurne Simón, Marta Molero-Luis, Ricardo Fueyo-Díaz, Cristian Costas-Batlle, Paula Crespo-Escobar, Miguel A. Montoro-Huguet

**Affiliations:** 1 GLUTEN3S Research Group, Department of Nutrition and Food Science, University of the Basque Country, 01006 Vitoria-Gasteiz, Spain; 2 Laboratory of Gastroenterology and Trace Elements, Department of Laboratory Medicine, Hospital Universitario La Paz, 28046 Madrid, Spain; marta.molero@salud.madrid.org; 3 PROSAM Research Group (S69-23R), Department of Psychology and Sociology, Universidad de Zaragoza, 50009 Zaragoza, Spain; rfueyo@unizar.es; 4 Department of Nutrition and Dietetics, Bradford Teaching Hospitals NHS Foundation Trust, Bradford BD9 6DA, UK; cristiancostasb@gmail.com; 5 ADViSE Research Group, Department of Health Science, European University Miguel de Cervantes, 47012 Valladolid, Spain; crespoescobar.paula@gmail.com; 6 Department of Nutrition and Obesity, Hospital Recoletas Campo Grande, 47007 Valladolid, Spain; 7 Gastroenterology, Hepatology and Nutrition Unit, University Hospital San Jorge, 22004 Huesca, Spain; 8 Department of Medicine, Faculty of Health and Sport Sciences, University of Zaragoza, 22002 Huesca, Spain; 9 Aragon Health Research Institute (IIS Aragon), 50009 Zaragoza, Spain

**Keywords:** celiac disease, malnutrition, nutrient metabolism gluten-free diet, nutritional deficiencies, nutritional assessment, dietitian, gluten immunogenic peptides, non-responsive celiac disease, psychologist, health resources, health costs

## Abstract

The gluten-free diet (GFD) remains a complex paradigm in managing celiac disease (CeD) in children and adults, and there are many reasons why GFD adherence should be strict to improve outcomes. However, this is a challenging task for patients, since they need to have access to quality healthcare resources that facilitate optimal GFD adherence. Understanding the strengths and weaknesses of the GFD, tackling coexisting nutritional deficiencies, and dealing with complex situations, such as seronegative CeD or non-responsive CeD, all require the involvement of a multidisciplinary team. The short- and long-term follow-up of CeD patients should preferably be performed by a combined Gastroenterology and Nutrition service with well-defined quality standards and the multidisciplinary involvement of physicians, nurses, dietitians, and psychologists. Nutritional advice and counseling by an experienced dietitian can reduce the costs associated with long-term follow-up of CeD patients. Likewise, psychological interventions may be essential in specific scenarios where implementing and sustaining a lifelong GFD can cause a significant psychological burden for patients. This manuscript aims to provide guidelines to improve clinical practice in the follow-up and monitoring of CeD patients and provide information on the nutritional risks of an ill-advised GFD. Clinicians, biochemists, food technologists, dietitians, and psychologists with a global view of the disease have been involved in its writing.

## 1. Introduction

Celiac disease (CeD) is an autoimmune disorder characterized by a systemic response to dietary gluten in genetically predisposed individuals, which has clinical manifestations of small bowel enteropathy associated with gastrointestinal as well as non-gastrointestinal symptoms [1,2,3,4,5].

The gluten-free diet (GFD) remains a complex paradigm in managing CeD. There are many reasons why it should be strict in order to improve outcomes. Firstly, voluntary or unintentional dietary transgressions remain the leading cause of persistent symptoms or enteropathy. Recent studies showed that even among patients who believed they were following a strict diet, a high proportion still had villous atrophy two years after starting the GFD, and many of them showed the presence of gluten immunogenic peptides (GIPs) in their stools [6,7]. Secondly, resolution of symptoms and enteropathy after gluten withdrawal is an essential criterion to confirm the diagnosis of seronegative celiac disease (SNCD) in patients carrying permissive genes (HLA DQ2.5, DQ8, DQ2.2. and DQ7.5), which is a recently acknowledged entity, especially in the adult population [8,9,10,11,12,13,14,15,16,17,18,19]. At this point, inadequate adherence to the GFD is a severe handicap for testing and verifying the diagnosis of SNCD [1,2,3,4,5]. Finally, a strict GFD is crucial to avoid developing long-term complications, such as anemia, osteoporosis, and malignancy [20]. A significant consideration is also the cost of investigating other clinical conditions often associated with CeD, which may also be responsible for persistent symptoms in individuals who claim to be strictly adhering to the GFD. These conditions can often include malabsorption of certain sugars (e.g., lactose, fructose, and sorbitol), intestinal bacterial overgrowth (SIBO), pancreatic exocrine insufficiency (PEI), microscopic colitis, Crohn’s disease, or irritable bowel syndrome (IBS) itself. Screening for these diseases is a costly burden for healthcare services, hence the importance of having expert registered dietitians (RDs) as an essential part of comprehensive celiac care units. Before considering these concomitant conditions, patients need expert RD advice and counseling, so inadequate GFD adherence can be ruled out before investigating other causes of symptoms.

Finally, mistakes in diagnosing CeD are still a relevant clinical problem that may result in patients being unnecessarily started on a GFD and this can also waste healthcare resources [14].

For all these reasons, the aim of this manuscript is to provide guidelines that will improve clinical practice in the follow-up and management of these patients. Towards this objective, we intend to provide information on the nutritional risks of a GFD diet and the consumption of gluten-free products, as well as delving into the different symptoms and nutritional deficits to be monitored, where the role of the registered dietitian is fundamental. In addition, the need for psychological support is highlighted. Finally, the economic advantages of this integrated approach are demonstrated. Clinicians, biochemists, food technologists, dietitians, and psychologists with a global view of the disease have been involved in drafting this manuscript.

## 2. Celiac Disease Nutritional Status before Diagnosis

### 2.1. Nutritional Status

The CeD patient generally presents with malabsorption, resulting from villous abnormalities in the small intestine and intestinal alteration, leading to multiple nutritional deficiencies [21,22]. The most frequently described deficiencies in children and adults with CeD at diagnosis are of iron, vitamin D, calcium, vitamin B12, folate and zinc [23]. Their prevalence is highly variable depending on many factors: age, a delayed diagnosis (i.e., the length of time the disease is active) and other modifying factors such as the extent of intestinal inflammation, the degree of malabsorption, and/or dietary intake [24].

Inadequate nutrient utilization can provoke a variety of nonspecific signs and symptoms, including faltering linear growth, short stature, poor weight gain in children, and weight loss in adults. Anthropometric data have indicated that those diagnosed in childhood tend to be underweight and short in length, and similarly, many celiac adult had a lower Body Mass Index (BMI) compared to the general population [25,26,27]. Being obese or, at least, presenting a high BMI has contributed to a delayed diagnosis of CeD by reducing its suspicion in the past [27], but it is currently common to diagnose CeD in overweight individuals [28,29,30].

### 2.2. Anemia and Iron Deficiency

Iron deficiency anemia (IDA) is one of the most recurrent extraintestinal manifestations in children and adults at diagnosis, being reported in over half of CeD patients (including subclinical CeD patients) [31]. Its prevalence is higher in adults than in children [32,33]. The primary cause is due to villous atrophy mainly occurring in the duodenum, the principal site of iron absorption. Anemia has been detected in almost 46% of cases with subclinical CeD, and it has been reported as the unique presenting feature in 39% of CeD patients [33]. One common pitfall is to attribute anemia to hypermenorrhea in a fertile woman as up to 4.5% of women with anemia and copious menstruation may have celiac disease [34]. Montoro-Huguet et al. pointed out that CeD patients with IDA at diagnosis have more advanced disease, slower dietary response, and worse recovery from mucosal lesions than those without anemia [35]. Latest studies suggest that up to 46% of cases are still iron-deficient one year after diagnosis. This may reflect a slow histological response related to dietary transgressions or blood loss through menstrual bleeding in women [31].

Regarding other micronutrients related to anemia, a lack of vitamin B12 is found in 8–41% of newly-diagnosed people, as its absorption mainly occurs in the distal ileum [24]. Although the etiology of vitamin B12 deficiency in CeD is unknown, potential causes such as decreased gastric acid, SIBO, autoimmune gastritis, or subtle dysfunction of the distal small intestine have been proposed [32]. Recent studies found a statistical association between the age group at diagnosis and the shortage of vitamin B12 in adults [36]. Shiha et al. described that compared to young adults, in whom impairment was around 10.5%, elderly celiac patients had a vitamin B12 deficiency of 20.2%, similar to other research reporting a 37% prevalence of this deficiency in people over 65 years of age [36].

Folate is primarily absorbed in the jejunum, and malabsorption is frequent in diseases of the small intestine [22,32]. Wierdsma et al. [30] found a 20% folate deficiency in people with untreated CeD, but this prevalence varies widely among studies [23,36,37,38,39,40]. Although studies carried out in the past highlighted the usual lack of folate in celiac patients [37], new research found no specific impairment in newly diagnosed individuals or, at least, any difference compared with CeD patients following a GFD [38].

### 2.3. Bone Health

Calcium absorption, like vitamin D, occurs in the small intestine, with higher rates in the duodenum [22]. The lack of calcium and vitamin D and subsequent metabolic bone diseases, such as reduced bone mineral density (BMD), are frequent co-morbidities in all age groups [41]. Approximately 75% of untreated adults with CeD suffer from low BMD and there are increased bone fracture risks in older people. In contrast, calcium deficiency during childhood and adolescence may cause growth problems and impair peak bone mass achievement [42]. This is particularly important, as metabolic bone disease may be the main manifestation of CeD in patients who do not exhibit gastrointestinal symptoms, thus leading to a delayed diagnosis of deleterious consequences [41,42,43,44]. A review [45] reported that most of its included studies found 25 (OH) vitamin D deficiency at CeD diagnosis, despite the assessed calcitriol being relatively high. This finding could be explained by the assumption that calcium malabsorption could result from reduced levels of calcium-binding proteins due to enterocyte loss, and not as a result of vitamin D deficiency. The impaired enterocytes can affect the response to calcitriol, further potentiating calcium loss and leading to secondary hyperparathyroidism [45], a finding of high prevalence in CeD patients [46].

### 2.4. Other Micronutrient Deficiencies’ Consequences

Other mineral deficiencies have been described at diagnosis of CeD, like zinc or copper [23]. It has been reported that one-third of children at diagnosis [40] and approximately two-thirds of untreated CeD patients were zinc-deficient [30]. This deficit is more related to an endogenous loss of zinc than this mineral’s malabsorption. Although clinical relevance remains inconclusive, reduced protein synthesis, cell-mediated immunity, antioxidant buffer capacity, diverse skin lesions and presentations associated with CeD may be ascribed partly to zinc deficiency [23,30]. Copper deficiency has been related to anemia and thrombocytopenia in both celiac adults and children [32], and also to neurologic impairment [47].

More recently, other mineral impairments have been attributed to the increased incidence of thyroid disease in patients with CeD [48,49], such as selenium [50] or iodine [51]. However, the involved mechanisms are not adequately established.

## 3. Following a Gluten-Free Diet

### 3.1. Advantages and Nutritional Risks

Currently, following a lifelong GFD is the only effective CeD treatment. Although notionally simple, the GFD has many complexities and it should be not only gluten-free, but also balanced, covering total energy and nutritional requirements [52].

Compared to a gluten-containing diet, several studies have revealed various nutritional deficiencies associated with following a GFD. Regarding macronutrients, the CeD population have a higher risk of insufficient fiber intake in both children and adults [26,53]. Churruca et al. [26] found that the mean fiber intake was 16.4 g/d in celiac women. This amount was significantly lower than that consumed by the control group of women and scarcely reached two thirds of the daily fiber recommendations. Children and adolescents with CeD have higher intakes of refined sugars and saturated fats due to the nutritional composition of gluten-free rendered products (GFPs) and the limited consumption of grains and unprocessed or unrefined cereal [54,55,56]. This low daily consumption of unrefined cereals also contributes to the low fiber content of the GFD. Regarding micronutrients, the GFD can lead to a lower intake of folate, iron, magnesium, selenium, niacin, biotin, riboflavin, pyridoxine, and vitamin D, among others [21]. 

In general, gluten-free raw food sources have fewer minerals such as iron, calcium, and magnesium, and industrial food processing may reduce the micronutrient content, which might be related to some nutritional deficiencies in CeD patients [57]. With regards to folate, for example, gluten-free flakes and pasta use ingredients such as corn and rice flours that have poor folate content compared to wheat flour. These alternatives may also include a variety of starches (potato, corn) that eliminate the protein-rich fractions of the flours, which can lead to a notable decrease in folate [26,56,58]. Similarly, this can occur with other mineral deficits found, which may also be caused by the lack of widespread fortification. While fortification of wheat flour is compulsory for some micronutrients, it is not mandatory for other alternative flours, such as those used in GFPs [26,56].

Furthermore, it is noteworthy that highly processed foods, such as GFPs based on grains and cereal, account for about 24% of the total energy intake in CeD children [56]. Apart from contributing to an imbalanced diet [56], recent studies indicate the risk of excessive GFP intake, and, for instance, consumption of ultra-processed foods is associated with inflammatory biomarkers in children with CeD [59]. Likewise, higher consumption of gluten-free bread, pastries, and sweet and salty convenience foods in the CeD adult population was associated with non-alcoholic fatty liver disease [60] (recently renamed with the term: metabolic dysfunction-associated steatotic liver disease) [61].

Recent studies have shown improvements in the formulation of GFPs [62], products whose market has grown exponentially. Compared to the past, an overview of the nutritional composition of GFPs reveals fewer differences with their gluten-containing counterparts, adding, e.g., more fiber and less salt. Nevertheless, GFPs usually maintain a poorer nutritional quality [62,63].

However, the nutritional imbalances described in the young and adult celiac population were also observed in healthy controls, both due to vitamin and mineral deficiencies and to excessive consumption of saturated fats and low consumption of unsaturated fats, linked to unhealthy dietary patterns in general [64,65]. Higher adherence to a Mediterranean diet has been associated with improved bone mineral density in CeD children [59].

A dietitian should deliver education on the prevention and management of the leading nutritional pitfalls of the GFD, to ensure a healthy and safe diet. Nutrition education provided by a dietitian to patients and their families can improve the nutritional profile of the GFD [66].

Nutritional counseling promotes the use of pseudocereals as substitutes for gluten-containing cereals because of their benefits in GFD. Amaranth and quinoa are good vitamin sources; sorghum contains a high level of thiamine and millet, and also provides carotenoids. These ingredients can improve the nutritional quality of GFPs, increasing protein, healthy fats, fiber, and minerals [67,68].

**Figure 1:**
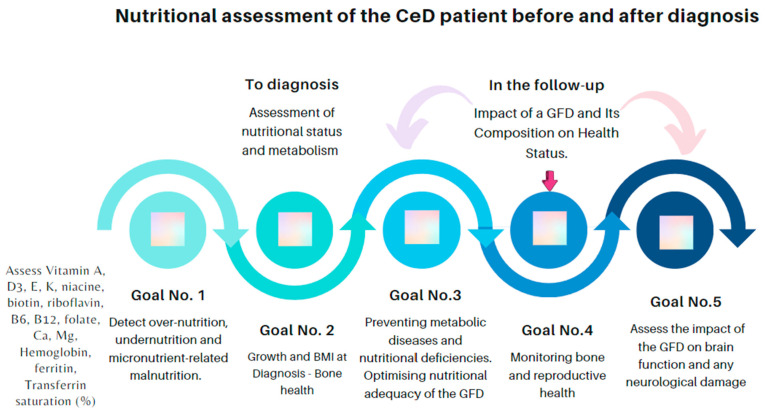
Remarks for clinicians and dietitians involved in the nutritional and metabolic care of CeD patients.

Recommendations for an overall healthy GFD should be similar to a regular healthy diet but with a focus on healthy alternatives to grain-based foods. In Europe, standard recommendations for a healthy diet include eating many fruits, vegetables, and complex carbohydrates and choosing foods lower in saturated fat, salt, and sugar. Additionally, it has been suggested to base the diet on the following frequency of consumption: every day, 2–3 portions of vegetables, 2–3 portions of fruits, 3–6 portions of gluten-free grains in CeD patients, 2 portions of milk and/or dairy, and 1–2 portions of protein sources (either animal or plant-based equivalent); every week, 5–7 portions of nuts and, at least, 1–2 servings of legumes [69]. Figure 1 shows the critical points in the nutritional assessment of the CeD patient at diagnosis and during follow-up, with an emphasis on the need to counterbalance the positive effects of gluten withdrawal with the nutritional deficiencies and potential risks resulting from poor dietary advice. In our environment we focus on the following points:

(1) Malnutrition, including over- and undernutrition, may be present in CeD, both at diagnosis and while under treatment. Underweight and growth retardation in children, which mostly reflect malabsorption, are not the rule. Nutritional deficiencies may be due to the poor absorption of amino acids and fats, as well as micronutrients, including calcium; iron; zinc; copper; vitamins A, D, E, and K; folate; and pyridoxine.
(2) Malabsorption of calcium and vitamin D with a chronic inflammatory state affects bone health and may result in osteopenia or osteoporosis in CeD. Thus, it is recommended to measure calcium, alkaline phosphatase, and vitamin D at CeD diagnosis to assess bone health. Moreover, it is necessary to perform bone mineral density measurement with the dual X-ray absorptiometry (DEXA) scan in adults, not later than the age of 30–35 years, especially if there is a history of fractures and growth retardation in childhood. Surveillance of nutritional status during follow-up.
(3) The nutritional composition of gluten-free rendered products (GFPs) can be unsatisfactory, and they are often not fortified with micronutrients. Therefore, the content of above-described vitamin and minerals in GFPs can be low, and these metabolic levels should be checked. In addition, eliminating gluten from the diet often impacts the proportion of nutrients consumed, leading to metabolic disorders. A critical point here is that GFPs are ultra-processed foods, which in the long term could be more detrimental to health. All this places the individual at risk for cardiovascular problems and metabolic-dysfunction-associated steatotic liver disease, the prevalence of which appears to be increased in this population when the gluten-free diet (GFD) has not been suitably advised.
(4) A GFD should be balanced with proper iodine, iron, calcium, and vitamin D, among others, advised by a dietitian.
(5) A GFD reduces or ameliorates some neurological symptoms, such as headache, ataxia, and epilepsy, in children and adults with CeD. In addition, a strict GFD can improve neuroimaging results in patients with gluten-sensitivity-related disorders.

### 3.2. Effects of the Gluten-Free Diet on Symptoms and Enteropathy

Several studies have supported that following a GFD leads to an improvement in clinical symptoms and normalization of antibodies in most people living with CeD. In contrast, intestinal lesions may persist for a longer time. Nevertheless, healing is affected by other factors such as age and gender: children heal at a quicker rate than adults, and men ameliorate more quickly than women, who also are more symptomatic than men. Other factors like occasional transgressions or low adherence to GFD and late onset of CeD with severe symptoms worsen the overall health status and recovery [70,71].

A strict GFD effectively alleviates classic gastrointestinal symptoms in the first year of treatment [72]. Many authors describe decreased abdominal pain and complete relief of bloating in weeks or months [72,73,74]. The period required for remission of other clinical signs, such as reflux and, mainly, diarrhea, varies substantially, and they may even persist after prolonged treatment [75]. These discrepancies could be due to the different definition criteria and the lack of mucosal restoration [72]. In some patients, persistent diarrhea is explained by other concomitant clinical conditions, including microscopic colitis (more frequent in CeD than in the general population), lactose or fructose intolerance, PEI, or SIBO. These entities should be intentionally sought out for in patients with persistent symptoms despite a strict GFD. 

Histological recovery of the small intestinal mucosa begins between 6 months and 3 years after initiating a GFD, allowing for sustained clinical response [76]. However, this statement is considered controversial. Studies conducted on children describe complete remission of histological lesions in 81.4% after at least a year of gluten withdrawal, or 97.6–100% in long-term GFD follow-up (≥three years) [76,77]. Conversely, the same authors established that 10.1% of celiac adults maintained villous atrophy after five years [76]. Rubio-Tapia et al. also found that only a third of patients (34%) achieved a mucosal recovery at two years on the GFD, whereas two thirds (66%) achieved it after five years on a GFD, even considering that a significant amount of them (82%) obtained clinical relief [71]. As mentioned above, a lack of intestinal recovery could be responsible for the persistence of one or more digestive signs in adults [78], especially in women. Research carried out by Dr. Fernández Bañares et al. (CADER study) [6] indicates that the rate of persistent villous atrophy after 2 years was high (53%) in adult patients with CeD on an intentionally strict gluten-free diet. Interestingly, 69% of patients in this series had detectable GIPs in at least one stool sample, strongly suggesting that low-level ongoing inadvertent gluten exposure could be contributing to persistent villous atrophy [6].

In addition, a lack of histological recovery has also been associated with IgA immunodeficiency [77]. Unfortunately for the patient, confirmation of histological recovery should only be performed using invasive techniques like a repeat endoscopy with duodenal biopsies, since others, such as serum anti-transglutaminase antibodies, are ineffective indirect markers [79,80].

Different molecules are proposed as possible surrogate non-invasive markers to predict intestinal damage, such as IgA anti-actin antibodies [78], serum intestinal fatty acid binding protein (I-FABP) [81] and plasma citrulline [82]. However, despite various studies conducted, these markers require further evaluation and validation.

Implementing GFD reduces other extraintestinal manifestations such as anemia, weight loss, or fatigue, which are all common before diagnosis. Anthropometric data such as BMI or low weight improve after removing gluten from children’s diets [83] or adults also living with CeD [27].

The incidence of anemia at the time of CeD diagnosis, mainly IDA, increases the more severe the intestinal villous lesion [35,84]. Annibale et al. [34] found that anemia was overcome in 77.8% of adults after 6 months on a GFD, and in 94.4% after 12 months, and this recovery was closely correlated with normalization of the intestinal mucosa. However, iron deficiency persisted in 50% of patients. Similarly, ferritin levels and folate were reduced [85]. It is important to note that severe villous atrophy (Marsh-Oberhuber 3c) can contribute to poor iron absorption, and in such cases, the repletion of iron stores may be very slow with oral iron. Some authors suggest that iron replacement should be carried out intravenously in these cases, as it resolves the symptoms of iron deficiency quickly, effectively, and safely, especially in adult patients with poor health-related quality of life [35]. Nestares et al. described a similar recovery time in celiac children diagnosed with anemia, who were prescribed oral iron supplements (ferrous sulfate, at doses of 3 to 5 mg per kilogram of weight per day). In addition, compared to a control diet, the GFD appears to be nutritionally less balanced and provided less iron and folic acid, among other micronutrients [84]. All of these factors can indeed make it challenging for CeD patients to optimize iron stores. 

Considering a recent review, fatigue at the time of CeD diagnosis has a mean prevalence of about 52% [86]. Though, this rate could be possibly higher if a specific fatigue instrument or tool were used. Published outcomes are limited probably because the experienced fatigue depends on many aspects, such as sleep disturbance, pain, and psychological conditions, such as depression, where the GFD may also help with symptom resolution.

### 3.3. Adherence to GFD

As previously mentioned, lifelong adherence to a strict GFD is the only treatment for CeD. However, a permanent strict GFD is not easy to achieve due to the presence of gluten in a vast number of products, gluten cross-contact of foods, inadequate food labeling, and social constraints [87,88,89]. Several studies have revealed that a considerable percentage of patients with CeD do not adhere to a GFD: rates for strict adherence range from 42 to 91%, depending on the definition and assessment method [90]. In addition, the difficulty in the assessing GFD adherence is increased by the interaction with several influencing factors, which need to be considered during a clinical evaluation (sociocultural background including parental education and job, age, gender, education, age of diagnosis, etc.) [91,92].

Considering age, and particularly children, mainly the group below 12 years of age comply best with the GFD, and most situations where GFD adherence is lacking tend to be due to unintentional causes. Conversely, adolescents are the most likely age group to intentionally not adhere to the GFD due to the difficulty of implementing it within their social environment [93,94]. In adulthood, men perform considerably more dietary transgressions than women, which may be explained by the fact that females suffer more symptoms, which can incentivize greater dietary self-control [95,96].

Finally, the clinical improvement in and resolution of symptoms achieved once the GFD is established after diagnosis tend to be signs of good adherence to the diet. At this point, Schlepatti A et al. [96] showed that good GFD adherence at the time of transition from pediatric to adult health care and “classic pattern” of presentation at the time of diagnosis were predictive of good long-term GFD adherence while being lost to follow-up was predictive of poorer long-term GFD adherence. These findings highlight the importance of identifying patients who may benefit during the transition phase to adult health care center so they can receive additional interventions to improve their long-term GFD adherence [93,96].

## 4. Patient Follow-Up

### 4.1. Tools to Monitor Adherence to the Gluten-Free Diet: Techniques and Procedures

Methods to monitor CeD were discussed in a recent ESPGHAN (European Society for Paediatric Gastroenterology Hepatology and Nutrition) position paper [97]. Several procedures involving various approaches to measure adherence to GFD can be found: (a) periodic visits by expert nutritionists, (b) structured questionnaires, (c) clinical follow-up, (d) CeD-specific antibodies, (e) GIP in stools and/or urine, (f) duodenal biopsies [91,97].

#### 4.1.1. Periodic Visits by Expert Dietitians and Dietary Records

Regular follow-up by CeD specialists increased adherence by 97.5%, compared to 40.4% in those who did not have this type of follow-up [98]. Protective factors for good adherence to the GFD include the direct care of multidisciplinary groups and the supervision of an expert dietitian. According to results from different studies, the measurement of GFD adherence through patients’ self-reports appears to be subjective and less accurate because it relies on the patient’s possibly limited knowledge of a GFD and gluten-free foods, as well as being time-consuming and having shown large variability [97,99].

Adherence to the GFD can be assessed by in-depth dietary interviews conducted by a trained dietitian and supplemented by dietary questionnaires, such as the Standardized Dietician Evaluation (SDE) [100,101]. However, there is considerable controversy about the validity of dietary questionnaires to assess a GFD because of some limitations, such as patients not intentionally recording actual gluten intake in the questionnaire or that this method is not standardized [102]. Other studies suggest that an expert dietitian’s intervention cannot help detect exposures in ~30% of patients with mucosal damage in duodenal biopsies [103].

Another issue lies in the fact that these assessments are non-objective and therefore not directly comparable, as different methods are used to check adherence, such as food diaries, 24 h recalls, dietitian interviews, self-reported questionnaires, food frequency questionnaires, or short questions [104]. Furthermore, there is no standard or quality control for dietary screening because the diets and habits of a particular population or region request a specific structured interview, which is linked to the quality of the diet [98]. Nonetheless, despite the absence of a gold standard to assess GFD adherence, a dietary evaluation by a trained dietitian is nowadays still considered one of the main methods of choice to determine adherence to a GFD [105,106]. 

When it comes to performing dietary records to assess gluten intake in CeD patients, there are currently no validated tools for this purpose. Therefore, it is advisable to choose a diet history that collects accurate information about patient food intake with relevant details (food preparation methods, ingredients of mixed dishes and recipes, amount of food consumed, and even the brand name of commercial products) [107,108]. In addition to the dietary record, the trained dietitian should ask about issues related to food, such as restaurants where the patient eats, food stores where food is bought, and any other matters related to gluten cross-contact to find out if the patient knows how to identify and avoid sources of gluten exposure [105,106].

#### 4.1.2. Structured Questionnaires

Over the last few years, several questionnaires to aid GFD adherence assessments on follow-up have been published. The Celiac Dietary Adherence Test consists of seven structured questions whose answers are measured on a Likert scale of 1–5 points, obtaining total scores of 7–35 points. A score of less than 13 indicates high adherence [109]. 

Alternatively, the questionnaire validated by Biagi et al. includes four questions ranging from 0 to 4. A score of less than three is considered low adherence to the GFD. It is designed to be a rapid and simple tool for checking commitment to GFD that could be used by personnel without specific CeD experience [110]. The Gluten-Free Diet Knowledge Scale (GFD-KS) questionnaire has also been used where participants were asked to classify a variable number of foods as “allowed”, “not allowed”, or “questionable”, and this allows to assess the dietary knowledge of people with CeD and/or their caregivers. The GFD-KS was developed by a panel of experts consisting of a gastroenterologist, a dietitian, and CeD patients [111]. In addition, some surveys are based, for example, on visual scales consisting of a line with “*I never adhere to the diet*” at one end and “*I always adhere to the diet*” at the other: or on a food frequency or self-reported adherence to the GFD questionnaire. These questionnaires, completed during a dietary review, can be used to detect unintentional gluten consumption, and can be performed by the responsible professional at that time. With regards to patients self-reporting adherence, patient subjectivity may arise in interviews with expert dietitians [98].

#### 4.1.3. Clinical Follow-Up: Recording of Symptoms

Clinical monitoring is a valuable and essential tool, particularly in patients who experience symptom improvement. The main challenges during CeD follow-up are to achieve the resolution of symptoms and recovery of intestinal damage [4].

There are various medical tools available for evaluating gastrointestinal symptoms in CeD. One example is the Celiac Symptom Index, which scores symptoms on a scale from 16 to 80. Scores of 30 or below are associated with good quality of life and adherence to a GFD, indicating clinical remission. By contrast, scores of 45 or higher are linked to poor quality of life and worse adherence to the GFD, suggesting active celiac disease [109]. Other tools include the Rome III diagnostic questionnaire, which utilizes a scoring algorithm [112], and the index Bristol scale [113]. 

Improvements in gastrointestinal symptoms are generally expected within days of starting the GFD [1]. However, abdominal bloating, pain, and constipation are the most common symptoms reported during follow-up [114]. Since strict adherence to GFD is highly effective in controlling symptoms and reducing healthcare utilization, experts recommend long-term monitoring of patients with CeD [1].

Recent research found that one-third of patients reported gastrointestinal symptoms or malabsorption signs when re-evaluated 12–28 months after beginning the GFD. Factors such as suffering symptoms for ≥5 years before CeD diagnosis or having constipation were associated with a higher odds ratio (5.3–95% CI 1.3 to 21.8- and 7.4–95% CI 1.3 to 42) [115]. However, it is well-known that there is no significant association between the persistence of GI symptoms and CeD serology or duodenal damage at histological re-evaluation [6,75,115], highlighting the low sensitivity of serology in predicting mucosal healing.

For pediatric celiac patients, normal growth and development are the primary goals. Multiple visits with a specialist physician or dietitian are recommended during the first year following diagnosis, with annual visits being a viable option after that [116].

The monitoring of CeD encompasses various approaches, including clinical follow-up and the use of other detailed tools mentioned in this article (Figure 1). The authors of this algorithm intend to highlight the importance of dietitians and psychologists in managing CeD patients.

#### 4.1.4. Serology: CeD-Specific Antibodies

Anti-tissue transglutaminase (TTG)-IgA is the main serologic marker in the diagnostic approach because of its high specificity and sensitivity, wide availability, and ease of adaptation in automated equipment. Several recent reports have demonstrated that TTG-IgA antibodies showed higher diagnostic accuracy than other serologic markers such as anti-gliadin [117,118,119] and anti-deamidated gliadin peptides (DGPs) antibodies [120]. In patients with IgA deficiency of any age, TTG, anti-endomysial (EMA), and DGP IgG isotype provide the best results due to their high diagnostic accuracy [112,121].

The strong association between elevated TTG-IgA values and Marsh II and III histopathological grades at the time of diagnosis has enabled to establish a cut-off (10 times to the upper limit of normal) that allows the diagnosis of CeD in pediatric patients without a need for duodenal biopsies [117,122]. This feature is a subject of debate in adults and is widely evaluated [122].

Follow-up of celiac patients will involve analysis of the TTG-IgA or the IgG isotype in case of patients with IgA deficiency. According to some studies, the median time to normalization of serology is 12 months from starting GFD in pediatric patients [123,124], with the TTG-IgA level at the time of diagnosis being the factor that most influences this recovery [124]. Thus, higher TTG-IgA levels at diagnosis were associated with a longer time to achieve serology normalization. Another independent predictor is poor GFD adherence, with age as the main influencing factor, where the lowest adherence levels are observed in adolescence [92,125].

According to the recent ESPGHAN recommendations [97], in pediatric and young adolescent patients, the first follow-up visit should be performed 3–6 months after CeD diagnosis and maintained every six months until TTG-IgA antibodies are at baseline values. After that, visits every 12 or 24 months are considered sufficient.

During follow-up, it is essential to use the same assay for serological determination, as there is currently no standardization of TTG-IgA antibodies, and the values obtained with different tests may vary significantly. This is because there are different methodologies to determine CeD antibodies in serum, such as enzyme-linked immunosorbent assay (ELISA), chemiluminescence (CLIA), and radioimmunoassay. The main difference among them relies on the detection type used (fluorescence, chemical reaction, or radioactivity). The CLIA methodology has remarkable sensitivity and specificity [123], which allows signal amplification. This is important to keep this in mind because TTG-IgA levels tend to persist longer and decrease more slowly over time compared to the ELISA method.

As several studies pointed out [97,112], the usefulness of serologic markers is still being determined. On the one hand, TTG-IgA and EMA-IgA analysis has a high negative predictive value when adult and pediatric patients have followed a GFD for one year or more [126]. On the other hand, the positive predictive value of these serological markers is low in adults and higher (approximately 0.70) in children [126]. An interesting study by Fang found that once TTG-IgA antibodies normalize, when TTG antibody values are below the analytical measurement range, they predict enhanced mucosal healing compared to those above detectable levels [127].

In summary, CeD-specific serological markers are inadequate to assess compliance to GFD and predict recovery of mucosal healing with certainty [97]), as they cannot detect small gluten exposures or intermittent gluten consumption [127]. 

In IgA-deficient patients, the same follow-up criteria are recommended as in IgA-sufficient patients, using the same CeD-specific IgG antibodies at diagnosis. However, it is noteworthy that IgG antibodies remained positive longer than TTG-IgA after GFD implementation [97]. In addition, many of these patients showed normal histology in their repeat biopsy regardless of antibody levels [128]. 

#### 4.1.5. Gluten Immunogenic Peptides (GIP) in Urine and Feces

Recently, a new tool has emerged to evaluate gluten intake and GFD adherence by determining the excretion of GIP in urine or feces. These peptides resist gastrointestinal digestion and appear to be involved in most immunotoxic reactions in T cells of patients with CeD [129]. They can be detected in stool [93] and urine through ELISA [130] and qualitative immunochromatography tests based on two different monoclonal antibodies (anti-33 mer a-gliadin peptide G12 and A1). Several clinical studies reported that GIPs provide high sensitivity and specificity for monitoring dietary adherence to GFD. Since the first study that found a strong correlation between urinary GIP concentrations and the degree of damage in the intestinal epithelium [129], many subsequent studies have reported similar results, not only in urine [131,132,133] but also in stools [6,93,134]. Furthermore, a strong correlation between the absence of GIPs in urine and the healing of the intestinal epithelium has been proposed [129]. 

In a longitudinal cohort study conducted in Sheffield, (UK) between 1998 and 2019, patients with established refractory celiac disease (RCD) type 1 and persisting mucosal inflammation and/or ongoing symptoms provided three urine samples for GIP analysis, and 17/36 (47.2%) had at least one positive urinary GIP test, suggesting that gluten exposure may be common in RCD type 1 [135]. In the CADER study [6], more than half of CeD patients supported to follow a strict gluten-free diet still showed villous atrophy two years later, and 69% of patients had detectable GIPs in at least one stool sample, strongly suggesting that low-level on-going inadvertent gluten exposure could be contributing to persistent villous atrophy [6].

Correct use of this method recommends analyzing multiple samples at different times or on different days, including both weekdays and weekends. It is convenient to collect two stool samples, for example, on Monday and Thursday, and three urine samples, such as two early morning samples and a final urine sample of the day. This approach is intended to achieve sensitivities and specificities above 95% [6,133,136].

Although this test has not yet been implemented in most national hospitals, it is important to note that some hospitals are now incorporating it into their laboratories, as several clinical studies recommend the utility of GIP in the management algorithm for CeD [134,137].

#### 4.1.6. Intestinal Biopsy

Since serological CeD markers have limitations in predicting mucosal healing, other tools, such as duodenal biopsies, must be considered. In general, guidelines agree that biopsies are not mandatory for patients without symptoms on a GFD [60].

In children and adolescents, intestinal biopsies are not indicated to assess mucosal healing [97]. It is only recommended to consider a repeat biopsy (or perform a biopsy for the first time) when there are doubts about the diagnosis of CeD, suspicion of additional diseases, or persistent clinical symptoms [127]. 

The requirement for a repeat biopsy is controversial in adults because it is unclear whether patients who respond to GFD and have negative autoantibodies should undergo a biopsy [127]. Some guidelines state that it is reasonable to perform a follow-up biopsy after 1–2 years on a GFD [4,98], especially in patients older than 40 years of age or in cases with a severe clinical presentation at diagnosis [137]. 

A duodenal biopsy is also mandatory to certify mucosal healing in patients with a well-founded clinical suspicion of CeD, negative serologic markers, and the presence of permissive genes for the development of CeD. Clinical practice guidelines state that this step is necessary to establish the diagnosis of SNCD reasonably [4,8,9,10,13,16,19,138].

Finally, the demonstration of intestinal mucosal healing in a patient with CeD whose symptoms persist despite good adherence to the GFD (e.g., with repeatedly negative stool or urine GIPs results) prompts investigation of other concomitant diseases that are truly responsible for the symptoms [139,140,141] (see below).

### 4.2. Non-Responsive Celiac Disease

Whilst most individuals diagnosed with CeD will display improvement in signs and/or symptoms after commencing a GFD, 7–30% of patients will continue to display clinical manifestations typical of CeD and/or have persisting intestinal inflammation [3,135,141]. When this occurs for at least 6–12 months despite adherence to a GFD, these individuals are classified as having non-responsive CeD (NRCD) [141]. In the setting of an apparent non-response to the GFD, the clinician should consider a wide spectrum of possibilities [142,143,144,145]:

1. The clinical condition responsible for the enteropathy is not true celiac disease and there has been a misdiagnosis In cases of doubt, re-evaluation of the biopsy specimen by one or two expert pathologists is necessary to exclude other causes of enteropathy including, small bowel bacterial overgrowth (SIBO), Whipples’s disease, Crohn’s disease, adult autoimmune enteropathy, common variable immunodeficiency, AIDS enteropathy, collagenous sprue, giardiasis, tuberculosis, or drugs (e.g., olmesartan), among others [1,3,4,10,15].
2. Once the initial diagnosis of CeD has been confirmed, the first step in the investigation of patients with ongoing symptoms is assessing for exposure to gluten. Patients will tend to overestimate their adherence to a gluten-free diet (GFD). The patient unintentionally or deliberately eats gluten or is extremely sensitive to minimal amounts of gluten (super sensitivity to gluten). In fact, some patients required as little as 10 mg of gluten per day to induce the development of intestinal mucosal abnormalities [135,146]. On the other hand, the persistence of symptoms is a severe problem in the presence of seronegative villous atrophy, where the resolution of symptoms and enteropathy is a mandatory requirement for diagnosis. In this context, dietary transgressions are an essential bias in interpreting the clinical course of these patients [12,16,138].
3. An associated pathology is the actual cause of the ongoing symptoms: microscopic colitis [147,148,149,150], SIBO [151,152,153,154], malabsorption of simple carbohydrates (e.g., lactose, fructose, or sorbitol) [155,156,157], and others such as reflux dysmotility [135], PEI [158,159], idiopathic bile salt malabsorption [160,161,162], Crohn’s disease, [163,164,165,166,167,168,169,170,171,172,173], and other functional digestive disorders, including irritable bowel syndrome [174,175,176]. In such patients, it is obvious that the nature of the symptoms is due to a different cause that overlaps with that of CeD itself. The active search for clinical conditions associated with CeD should be based on a judicious and cost-effective clinical assessment. For example, microscopic colitis must be ruled out if the dominant symptom is watery diarrhea in a woman who smokes cigarettes and takes regularly nonsteroidal anti-inflammatory drugs or omeprazole. Likewise, if the predominant symptom is flatulence and abdominal distension, or “explosive diarrhea” (mixed with abundant gas), it is a priority to rule out carbohydrate malabsorption, SIBO, or both. Finally, the GFD is often low in fiber, which may exacerbate constipation causing pain and bloating symptoms. Many patients with CeD will also fulfil the Rome IV criteria for irritable bowel syndrome (IBS) and in cases where other causes of symptoms have been excluded, this may be the most likely cause
4. The patient developed RCD. Once other causes of ongoing symptoms have been ruled out, a diagnosis of true refractory celiac disease (RCD) can be considered. True RCD is a rare condition defined as persistent malabsorptive symptoms and villous atrophy despite strict adherence to a GFD with negative serology for anti-tissue transglutaminase or anti-endomysial antibodies; [135].
5. Rarely, persistence or worsening of symptoms may be due to the development of serious complications such as ulcerative jejunoileitis or malignancy (e.g., enteropathy-associated T cell lymphoma or small bowel adenocarcinoma) [135,140].

**Figure 2:**
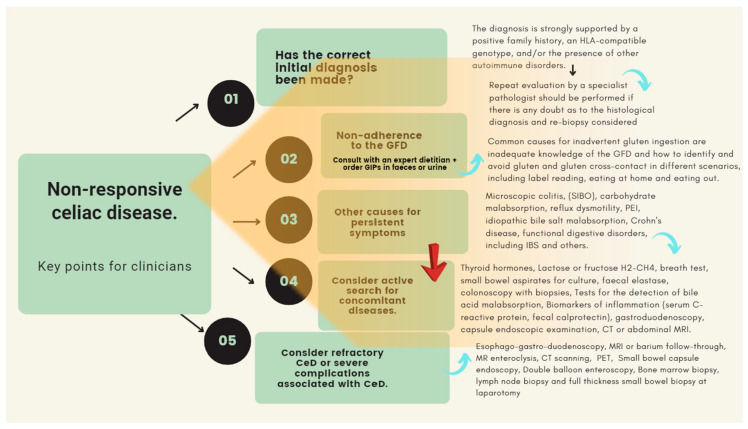
Approach to a patient with non-responsive CeD. Abbreviations: CT: computed tomography; MRI: magnetic resonance imaging; PET: positron emission tomography; PEI: pancreatic exocrine insufficiency.

Exploring the root cause of symptoms for these patients is complex, involves significant healthcare costs, and requires an expert approach [9,10,15,141,177]. Figure 2 shows a structured approach to support this group of patients.

## 5. Psychological Considerations and Time-Related Variables

CeD can be burdensome and limiting, especially in those patients with associated symptoms. Four time-related variables could affect adherence to GFD and its psychological impact: first, the patient’s age—how an adolescent experiences CeD may differ from how an older adult experiences CeD and the GFD. Second, age at diagnosis—being diagnosed in childhood may vary from being diagnosed during adulthood. In other words, both current age and age at diagnosis play a role in how CeD impacts the patient. Third, time to diagnosis—for example, experiencing seven years of debilitating symptoms before reaching a diagnosis is different to being diagnosed within the first year after developing symptoms). Finally, time dealing with GFD—the longer your experience dealing with the GFD, the easier it might be. It is worth noting that adherence to GFD usually leads to an improvement in quality of life (QoL), except for cases where there are challenging socio-economic factors, or in cases of asymptomatic patients that must assume GFD inconveniences without noticeable benefits, as they already had a good QoL.

In children on a GFD, a systematic review [90] found that different studies suggest an improved QoL. Still, the results achieved only significance in a single study [178]. Children seem to have a better health-related quality of life (HRQoL) than adults but children with asymptomatic CeD do not see QoL improvements after diagnosis [4]. A recent metanalysis [179] reported no differences in HRQoL between children with CeD and healthy control groups. In addition, parents perceived lower HRQoL in their offspring than that perceived by the children themselves. Two out of four studies showed a negative relationship between age at diagnosis and HRQoL. Therefore, the younger an individual at diagnosis, the more likely they are to manifest a higher quality of life.

Qualitative research reports social isolation, fear of being forgotten, and lack of spontaneity which requires constant planification as the main psychological symptoms associated with CeD and the GFD [180]. Some children may deal with these situations with lower anxiety when provided with coping strategies to increase their confidence [181].

Studies involving CeD adolescents often appear together with children, making it difficult to show the results stratified by age. Food-related situations at school, at home, or eating out are factors that negatively impact their emotions and social relationships and may affect their daily routines [97]. These situations convey feelings such as sadness when dining or anger for having to be on a GFD. Once again, and especially in adolescence, the time of diagnosis and their experience with GFD may play a mediating effect on the impact the GFD can have.

In adulthood, CeD can be very limiting, mostly in patients that do not follow a GFD and experience symptoms such as abdominal discomfort and signs of malnutrition [182,183,184]. Starting a GFD usually improves QoL, but if it does not, it might be due to the social restrictions that the GFD involves [183]. Establishing a GFD in asymptomatic patients diagnosed through proactive screening can deteriorate their QoL [185,186]. Recent findings showed depression and anxiety were present in 50% of newly diagnosed patients [45]. Women seem to present a higher risk of associated anxiety despite following a GFD. These symptoms might mitigate once experience is gathered in the management of the GFD.

In a recent study [187], Fueyo-Díaz et al. analyzed the impact of adherence to GFD on QoL using the Spanish version of the SF 12-item (Short Form Health Survey). A low QoL was not evidenced, but instead, significant differences were found between the physical and mental components, the latter being lower. Using CeD quality of life (CDQoL) survey, in this same study, HRQoL was high (M = 72, 73; SD = 16.83), above the cut-off point of 70 which determined a good HRQoL. When analyzing the subscales of the questionnaire, it was “health concerns” and “limitations” where lower scores were obtained. A remarkable 38.8% of participants had a total score below 70, showing a low HRQoL measured with the CDQoL. By age group, they reported significant differences between age groups (18–35, 36–50 and above 50), thus finding a direct relationship between age and HRQoL. Consequently, the older an individual, the higher the quality of life tends to be.

Qualitative research found that a restrictive diet, being constantly on alert and unwanted visibility at social events were the most reported signs of discomfort when dealing with GFD [187,188]. Celiac patients reported limitations when eating out, constant worry about gluten, continuous planning, feeling different, emotional pressure, or coping with symptoms. Right after diagnosis, many patients feel anger, fear, shame, rage, and grief, but after some time in GFD, the situation normalizes, and their HRQoL improves [189].

The diagnosis of CeD in the elderly has increased significantly since 1998, and research has shown that they experience less intense gastrointestinal symptoms, such as diarrhea and gut pain, than younger patients [23]. In the symptomatic elderly, adherence to GFD has demonstrated to improve symptoms and quality of life [190]. These ameliorations are maintained after two years of follow-up [191]. Nevertheless, there are few studies exploring psychological symptoms in older people with CeD, and soon, quantitative, and qualitative research will be needed to further explore CeD in this age group.

In summary, providing coping tools (such as social skills, acceptance, and control) and social support through a psychologist are the most important strategies to mitigate the impact of CeD at any age, including developing adequate awareness for optimal long-term adherence to the GFD. 

## 6. Cost-Efficacy of Optimal GFD Adherence Monitoring

Since the GFD is the only available treatment for CeD [135], it is essential that GFD adherence is adequately monitored and that patients are educated thoroughly to ensure they know how to avoid gluten correctly. Strict GFD adherence can help to control symptoms, improve quality of life, and decrease the risk of complications [192]. In addition, adequate GFD adherence can also effectively reduce healthcare utilization [193]. However, a recent systematic review reported that rates of adherence to a GFD ranged between 45 and 90% in patients of different ethnicities living with CeD [88], thus highlighting how GFD adherence can be notably variable. The main factors identified to influence GFD adherence in the review were age at diagnosis, coexisting depression, symptoms on ingestion of gluten, nutrition counselling, knowledge of GF foods, understanding of food labels, cost and availability of GF foods, receiving GF foods on prescription and membership of a celiac society. When facilitators and barriers to the GFD have been further explored through a systematic review that included forty studies [86], lower knowledge of CeD, restaurant and/or supermarket shopping, and poor patient education from practitioners were identified as the most significant barriers. Conversely, the most significant facilitators included:

- Increased education.
- Increased knowledge of a GFD.
- Increased intention/self-regulatory efficacy.

Therefore, adequate GFD education and knowledge transfer appear to be critical components when it comes to supporting patients with GFD adherence.

Dietitians have an important role in helping people achieve GFD adherence through education and knowledge transfer. This is because they are trained to assess patient knowledge, dietary adherence, nutritional status, psychosocial needs, monitor laboratory results, and provide individualized dietary advice [67]. Moreover, a skilled dietitian assessment is considered the non-invasive gold standard for GFD adherence, as it can identify if there are inadvertent sources of dietary gluten [99,194,195]. This can be useful in many clinical settings, but particularly where patients may return to clinicians with persisting symptoms and problems. A wealth of evidence suggests that the most common cause of persistent symptoms in CeD is ongoing gluten ingestion [135,142,196,197], and this is likely why current American CeD guidelines suggest that patients with non-responsive CeD receive an expert dietitian evaluation before considering a repeat small bowel biopsy [1]. If gluten ingestion is identified by the dietitian and patients are educated on how to avoid gluten and symptoms resolve, further investigations are likely to be avoided, which can save significant healthcare resources.

In the UK, the dietitian’s role in supporting patients living with CeD has been explored using dietitian-led celiac clinics, where specialist dietitians become the leading healthcare provider supporting patients living with CeD, with support from a gastroenterologist when needed. A recent UK service evaluation [198] of a newly implemented dietitian-led celiac service found that it was associated with a significant reduction in both voluntary and involuntary gluten ingestion after all 170 patients had received dietary education from a specialist CeD dietitian. GFD adherence improvements happened despite patients being previously followed-up by a gastroenterologist or celiac nurse in the same hospital. Furthermore, despite 104 patients self-reporting strict GFD, the specialist dietitian assessment identified that 39% were still eating gluten involuntarily. This created an opportunity to further educate patients about GFD adherence, and involuntary gluten ingestion was significantly reduced to 15% on follow-up (*p* < 0.001). In addition, nine patients with persistent problems were referred by gastroenterologists to the specialist dietitian for a GFD adherence assessment before considering arranging a repeat endoscopy with duodenal biopsies. All nine patients were identified to be eating gluten by the dietitian. GFD adherence education was provided, and symptoms resolved on follow-up, meaning that nine repeat endoscopies were avoided, quantified as GBP 3780 savings by the authors. Despite this being an observational study, it highlights scenarios of clinical practice where specialist dietitian input can help patients improve GFD adherence and reduce unnecessary investigations. In addition, the study also highlighted that more input from specialist dietitians could result in less need for gastroenterologist appointments when there are no active medical issues. This cost saving was not quantified. However, another UK dietitian-led celiac service evaluation [199] identified that a follow-up appointment with a dietitian could cost around half of what a gastroenterologist or nurse follow-up appointment costs (GBP 54 vs. GBP 110). Thus, offering optimal GFD adherence monitoring has a notable and worthwhile impact on health service cost-savings. 

More studies are needed to define the safety, efficacy, cost-saving opportunities, and patient acceptability of dietitian-led celiac clinics. However, having more follow-up appointments with a specialist dietitian and having a dietitian as one of the main health professionals involved in patient’s care are likely to be well accepted by patients. This is because in a previous UK study that enquired about patient preferences with regards to follow-up, the most preferred method of CeD follow-up was to see a dietitian with a doctor being available when needed [200], and another recent UK service evaluation has replicated these findings [201].

Overall, monitoring of the GFD through a dietitian with adequate expertise in CeD can ensure patients are educated thoroughly and promptly with regards to following a GFD so that more expensive appointments with a doctor or more costly and invasive investigations like repeat duodenal biopsies can be used less frequently and only when truly necessary.

## 7. Follow-Up Algorithm in CeD

**Figure 3:**
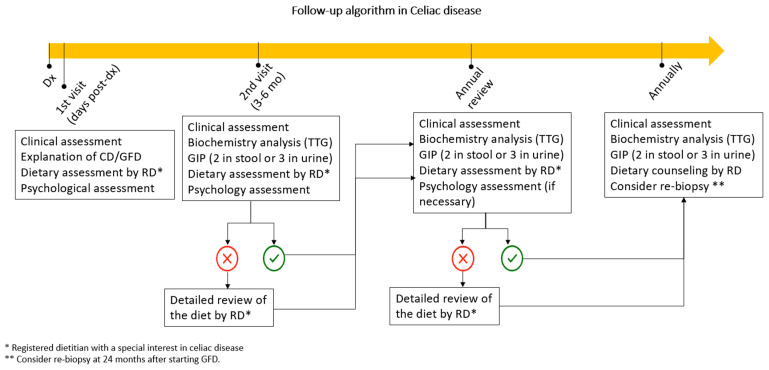
Follow-up algorithm in CeD.

Figure 3 shows the follow-up algorithm postulated in the present work. After diagnosis, a first multidisciplinary visit with the patient is advisable to explain the disease and the GFD adequately. During this visit, intervention with a psychologist is also necessary to avoid the anxiety that the newly diagnosed condition and change in lifestyle can sometimes produce. After 3–6 months of treatment, clinical and biochemical evaluation (including GIP), dietary counseling, and psychological support may be recommended. The annual visit, after diagnosis, would include these same sections where the dietitian and psychologist could address any chronicity since there have already been outings to restaurants, vacations, and experience in various psychosocial situations. The inclusion of the psychologist in the annual visit will be established on an as-needed basis.

## 8. Gluten-Free Diet in CeD: Conclusions and Highlights

In CeD, gluten triggers an immune reaction leading to enteropathy, malabsorption, and symptoms. A strict GFD is the basis for managing these patients. The following five postulates should be in the minds of healthcare professionals in charge of these patients.

1. In comparison with gluten-containing food, gluten-free alternative grains can render a lower amount of protein, dietary fiber and certain vitamins and minerals. Excessive consumption of ultra-processed foods such as many GFPs could have negative effects on health due to their inadequate nutritional composition (e.g., high sugar and saturated fat content).
2. Following a GFD also entails significant challenges beyond avoiding gluten. It is also essential to correct nutritional deficits and achieve dietary balance. In addition, substituting manufactured GFPs with naturally gluten-free alternatives is essential to reduce the risk of metabolic disorders associated with high consumption of ultra-processed foods. In both cases, the role of a dietitian with specific training and expertise in this area is essential.
3. Nutritional advice and counseling by an experienced dietitian can reduce the costs associated with long-term follow-up of the CeD patient. It should be noted that non-responsive CeD is a complex entity in which numerous causes may be involved (see Figure 2). Its investigation can be time-consuming, challenging, and cumbersome. In this regard, it should be borne in mind that inadequate adherence to the GFD remains the most frequent cause.
4. Celiac patients report difficulties when eating out, constant worry about gluten, continuous planning, feeling different, emotional pressure, or coping with symptoms. Right after diagnosis, they feel anger, fear, shame, rage, and grief, but after some time in GFD, the situation normalizes, and their HRQoL improves. Psychological interventions are crucial in these scenarios.
5. For all the above reasons, the short- and long-term follow-up of celiac patients should preferably be performed by a gastroenterology, hepatology, and nutrition unit with well-defined quality standards and the multidisciplinary involvement of physicians, nurses, dieticians, and psychologists (see Figure 3). This approach has been shown to reduce the costs associated with their health care.

## Data Availability

Not applicable.

## Abbreviations

The following abbreviations are used in this manuscript: CeD: celiac disease; GFD: gluten-free diet; GIPs: gluten immunogenic peptides; SNCD: seronegative celiac disease; SIBO: intestinal bacterial overgrowth; PEI: pancreatic exocrine insufficiency; IBS: irritable bowel syndrome; RD: registered dietitian; BMI: Body Mass Index; IDA: iron deficiency anemia; BMD: bone mineral density; GFP: gluten-free rendered products; ESPGHAN: European Society for Paediatric Gastroenterology Hepatology and Nutrition; GFD-KS: Gluten-Free Diet Knowledge Scale; Ig: immunoglobulin; TTG: anti-tissue transglutaminase; EMA: anti-endomysial; DGP: anti-deamidated gliadin peptides; ELISA: enzyme-linked immunosorbent assay; CLIA: chemiluminescence; RCD: refractory celiac disease; NRCD: non-responsive celiac disease; QoL: quality of life; HRQoL: health-related quality of life; CDQoL: celiac disease quality of life; CT: computed tomography; MRI: magnetic resonance imaging, PET: positron emission tomography.
